# Supplementary material for: Phrenic-specific transcriptional programs shape respiratory motor output
Source: eLife. 2020 Jan 16;9:e52859. doi: 10.7554/eLife.52859 (PMC7007220; doi:10.7554/eLife.52859)
Supplement: Supplementary file 1. — Supplementary Table 1. PCR primers for in situ hybridization. Supplementary Table 2. Specificity of in situ probes for PMC cadherins. To ascertain the specificity for the designed in situ probes to its intended target (given the degree of similarity between members of the cadherin family), we ran each full length probe through BLAST (https://blast.ncbi.nlm.nih.gov/) against the refseq_rna database for M. musculus and checked for cross-reactivity with other cadherins. BLAST outputs a ‘max alignment score’ parameter, which awards points for base matches while penalizing base mismatches, gaps, etc. While all on target alignment scores are high (>1400, dependent on probe length), off target scores are low (<35% of the max alignment score of the intended target). [file elife-52859-supp1.docx]

**Supplementary File 1**

| **Name** | **Forward** | **T7 + Reverse** | **Product Size** |
| --- | --- | --- | --- |
| Cdh1 | CCCAGAGACTGGTGCCATTT | TAATACGACTCACTATAGGGTTTCGAGTCACTTCCGGTCG | 747 |
| Cdh2 | ACAGCGCAGTCTTACCGAAG | TAATACGACTCACTATAGGGGGCCATAAGTGGGATTGCCT | 963 |
| Cdh3 | CAACGAAGCCCCTGTGTTTG | TAATACGACTCACTATAGGGCGTTTCGGAGAACCACCTCA | 899 |
| Cdh4 | ACACAAGAAAGGGCAGACAG | TAATACGACTCACTATAGGGCTGTTGCTACAGTGCCTGCT | 609 |
| Cdh5 | TTGGACCGAGAGAAACAGGC | TAATACGACTCACTATAGGGAGGGTTACCCCGAGAATCCA | 729 |
| Cdh6 | CCAGCCCTACCCAACTTTCT | TAATACGACTCACTATAGGGCCTTCCTGGGTTTCCTGGTC | 939 |
| Cdh7 | GGGAAGCTCCACTCTGATGT | TAATACGACTCACTATAGGGTGTGTCGCTTCTGACACCTC | 968 |
| Cdh8 | AGGTTTTCCATTGACCGCCA | TAATACGACTCACTATAGGGAATCCCCACCGGGGTAAAAA | 824 |
| Cdh9 | ATGTAGATGAGCCCCCTGTG | TAATACGACTCACTATAGGGCCTCTTCAATGCAGCAAACA | 788 |
| Cdh10 | TGACCCTTCCTATGGAAACA | TAATACGACTCACTATAGGGCCACCTAAGGGGTCATCTTT | 999 |
| Cdh11 | CTATACCACAGCCAGGCGTT | TAATACGACTCACTATAGGGATCTGGTACACGCTCTGTGG | 776 |
| Cdh12 | AGCCCCAACAGACTTTAGCC | TAATACGACTCACTATAGGGGAGCCAACAGGGGAAGACTC | 781 |
| Cdh13 | CCGAGAACTCCGCTCACC | TAATACGACTCACTATAGGGCAAACGCTGTCATCCGCATC | 805 |
| Cdh15 | AGCCAGATTAACGTGAGCCA | TAATACGACTCACTATAGGGTGAGAGCTGTGTCGTAGGGA | 610 |
| Cdh16 | TTACCTCTCTGTTATTCAGCTACCA | TAATACGACTCACTATAGGGCTGGTAGTGCACGTCTCCTC | 703 |
| Cdh17 | GAAGGCCAAGAACCGAGTCA | TAATACGACTCACTATAGGGAGACATGTCGGTGGGTTGTC | 905 |
| Cdh18 | ATCCACCTGAACTTGCCAGG | TAATACGACTCACTATAGGGTACGTGAACTCCTCTGCTGC | 601 |
| Cdh19 | ACAAGCTACTGGGGATTGGC | TAATACGACTCACTATAGGGGTGGAAGCGTTAACATGGGC | 823 |
| Cdh20 | ACTGTGAAGAAGCCCCTGAG | TAATACGACTCACTATAGGGCTTGAATGGTAAGCGTGCCG | 775 |
| Cdh22 | AGGAGTACACGGGGACAGAA | TAATACGACTCACTATAGGGACTCAAAGTCCAGGTGCTTC | 831 |
| Cdh23 | TGCGCAGACAGAACTGCTTA | TAATACGACTCACTATAGGGTTCAGCAGCGAGCCCTTAAT | 636 |
| Edil3 | AAATGGTGGCATCTGTCTGT | TAATACGACTCACTATAGGGCATTTCCACGAAAGACCATC | 721 |
| Ptprt | CACCACCTCTTTGTGGGTCT | TAATACGACTCACTATAGGGCACTCTGGGTCTCCTTCTGC | 700 |
| Lsamp | ACTCAAAAGTGGCCTGGTTG | TAATACGACTCACTATAGGGGGATCCGTTGATTCCTCTCA | 776 |
| Negr1 | AGCGGTGCTCAGGTGTTACT | TAATACGACTCACTATAGGGTTGGTCACTGTGAGGATGGA | 693 |

**Supplementary Table 1.** PCR primers for *in situ* hybridization

**Supplementary Table 2.** Specificity of *in situ* probes for PMC cadherins

| **Name** | **Product Size** | **% of probe in**  **extracellular**  **domain** | **On target BLAST max score** | **Off target BLAST max score #1** | **Off target BLAST max score #2** | **Off target BLAST max score #3** |
| --- | --- | --- | --- | --- | --- | --- |
| Cdh2 | 963 | 100 | 1737 | 312 (cdh4) |  |  |
| Cdh6 | 939 | 100 | 1694 | 570 (cdh9) | 568 (cdh10) | 374 (cdh20) |
| Cdh9 | 788 | 91 | 1422 | 279 (cdh6) | 265 (cdh10) |  |
| Cdh10 | 999 | 100 | 1802 | 428 (cdh6) | 376 (cdh9) | 333 (cdh12) |
| Cdh11 | 776 | 100 | 1400 | 408 (cdh20) | 347 (cdh24) | 331 (cdh8) |
| Cdh22 | 831 | 100 | 1499 | 232 (cdh24) | 229 (cdh20) | 226 (cdh12) |

To ascertain the specificity for the designed *in situ* probes to its intended target (given the degree of similarity between members of the cadherin family), we ran each full length probe through BLAST (<https://blast.ncbi.nlm.nih.gov/>) against the refseq_rna database for *M. Musculus* and checked for cross-reactivity with other cadherins. BLAST outputs a ‘max alignment score’ parameter, which awards points for base matches while penalizing base mismatches, gaps, etc. While all on target alignment scores are high (>1400, dependent on probe length), off target scores are low (<35% of the max alignment score of the intended target).
